# Biomechanical Comparison of Three Fixation Constructs for Tile Type C1.2 Pelvic Ring Fractures: A Finite Element Analysis

**DOI:** 10.3390/life16020336

**Published:** 2026-02-15

**Authors:** Adrian Claudiu Carp, Bogdan Veliceasa, Dmour Awad, Alexandru Filip, Mihaela Perțea, Norin Forna, Bogdan Puha, Ștefan Dragoș Tîrnovanu, Mihnea Theodor Sîrbu, Silviu Dumitru Pavăl, Paul Dan Sîrbu

**Affiliations:** 1 Department of Orthopaedics and Trauma/Surgical Science (II), Faculty of Medicine, Grigore T. Popa University of Medicine and Pharmacy Iasi, 16 University Street, 700020 Iasi, Romania; adrian-claudiu.carp@umfiasi.ro (A.C.C.); dmour-awad@umfiasi.ro (D.A.); alexandru-filip@umfiasi.ro (A.F.); norin.forna@umfiasi.ro (N.F.); bogdan.puha@umfiasi.ro (B.P.); stefan-dragos.tirnovanu@umfiasi.ro (Ș.D.T.); mihnea-theodor.sirbu@umfiasi.ro (M.T.S.); paul.sirbu@umfiasi.ro (P.D.S.); 2 Department of Plastic Surgery and Reconstructive Microsurgery, Surgical Science (I), Faculty of Medicine, Grigore T. Popa University of Medicine and Pharmacy Iasi, 16 University Street, 700020 Iasi, Romania; mihaela.pertea@umfiasi.ro; 3 Department of Computer Science and Engineering, “Gheorghe Asachi” Technical University of Iasi, 27 Dimitrie Mangeron, 700050 Iasi, Romania; silviu-dumitru.paval@academic.tuiasi.ro

**Keywords:** pelvic ring fracture, Tile type C1.2 fracture, finite element analysis, biomechanics, iliosacral screw, transiliac plate, anterior reconstruction plates

## Abstract

Fractures of the pelvic ring are among the most severe injuries in orthopaedic practice and Tile type C lesions are characterized by complete disruption of the posterior arch with both vertical and rotational instability. The optimal construct for posterior ring fixation remains a matter of debate. The aim of this study was to compare, by means of finite element analysis, the biomechanical performance of three different methods of osteosynthesis for Tile type C1.2 pelvic ring fractures: a transiliac plate, one iliosacral screw and two anterior reconstruction plates on the sacroiliac joint. A three-dimensional model of an intact pelvis was reconstructed from computed tomography images of a healthy adult male. A Tile type C1.2 injury pattern was created virtually, and three fixation constructs were designed in Ansys SpaceClaim according to manufacturer specifications. All materials were assumed to be homogeneous, isotropic and linearly elastic. Vertical loads of 400 N and 800 N were applied to the sacral endplate to simulate partial and full weight bearing, while the acetabular regions were constrained to represent standing stance. In this study, mechanical stability was operationally defined as resistance to global displacement under applied vertical load, with lower displacement indicating higher construct stiffness. Construct stiffness, total deformation and von Mises stress were assessed for bone and implants. For both loading conditions, the iliosacral screw construct showed the lowest overall displacement and provided the greatest stiffness. The transiliac plate construct presented larger displacements, whereas the anterior reconstruction plate construct provided intermediate stability with higher stresses at the sacroiliac joint. Among the analyzed constructs, the iliosacral screw provided the greatest stiffness and lowest overall displacement, suggesting superior mechanical performance under vertical loading conditions.

## 1. Introduction

Fractures of the pelvic ring are among the most severe injuries in orthopaedic practice, accounting for 2–8% of all skeletal injuries [1]. Tile type C fractures involve complete disruption of the posterior pelvic ring and are characterized by both vertical and rotational instability [2]. Non-surgical management of this type of fracture is associated with immediate and long-term complications such as chronic pain, abnormal gait, prolonged bed rest and lower limb length discrepancy [3]. These sequelae significantly impair quality of life in a predominantly young and active patient population.

To restore anatomical integrity, Tile type C fractures generally require combined anterior and posterior stabilization, with posterior fixation representing the cornerstone of re-establishing pelvic ring stability [4]. The current gold standard for anterior stabilization is open reduction and internal fixation with plate. In contrast, several competing options exist for posterior fixation and the optimal construct remains controversial [5]. Iliosacral screws are widely used because they can be inserted percutaneously through small incisions, with a reduced risk of infection and soft-tissue complications when compared with open techniques [6,7,8,9,10]. Alternative strategies include posterior fixation with a transiliac plate, which offers the advantage of a low rate of neurovascular injuries [11], and anterior reconstruction plates across the sacroiliac joint, which permit direct visualization of the reduction during the procedure [5].

Finite element analysis (FEA) has become an increasingly useful tool for comparing different fixation strategies in a controlled environment without exposing patients to additional risk. By simulating physiological loading conditions and quantifying stress and displacement, FEA provides insight into construct stiffness and potential failure zones that may not be readily apparent in clinical series alone.

Despite several existing biomechanical studies on posterior pelvic fixation, direct comparisons between iliosacral screws, transiliac plates, and anterior sacroiliac reconstruction plates in unilateral Tile C1.2 fractures remain lacking. For example, Hu et al. [12] compared three posterior fixation strategies using finite element analysis and reported differences in implant stress and stress shielding, while Song et al. [13] examined various pedicle screw configurations versus plate fixation in Tile C fractures, demonstrating that screw number, diameter, and placement significantly affect stability. However, based on current literature, no study to date has directly evaluated these three specific constructs within a standardized Tile C1.2 fracture model.

The aim of this study was to compare the biomechanical performance of three different methods of osteosynthesis used for the fixation of Tile type C1.2 pelvic ring fractures, namely a transiliac reconstruction plate, one iliosacral screw and two anterior reconstruction plates on the sacroiliac joint, using three-dimensional finite element analysis.

## 2. Materials and Methods

### 2.1. Geometry Reconstruction

The three-dimensional geometry of the pelvis was reconstructed from computed tomography images of a 30-year-old male subject, 180 cm tall and weighing 85 kg, who had no previous pelvic pathology and agreed to the use of his anonymized data for research purposes. The CT scan (Siemens Somatom, Siemens Healthineers, Erlangen, Germany) was acquired in the transverse plane with a slice thickness of 0.6 mm and a pitch factor of 0.8. DICOM data were imported into Ansys SpaceClaim 2024 R1 (Windows) (ANSYS Inc., Canonsburg, PA, USA) and semi-automatic segmentation was used to isolate the bony structures of the pelvis. The segmentation process included threshold-based isolation of bony structures followed by manual refinement of cortical boundaries. Cortical bone was not modeled as a separate shell layer; instead, the pelvic bone was treated as a solid body with uniform material properties. Sacroiliac joint cartilage and ligamentous structures were not included in the model. The model is intended to represent a typical adult pelvic anatomy for comparative analysis and is not meant to reflect the full anatomical variability of the general population.

**Figure 1:**
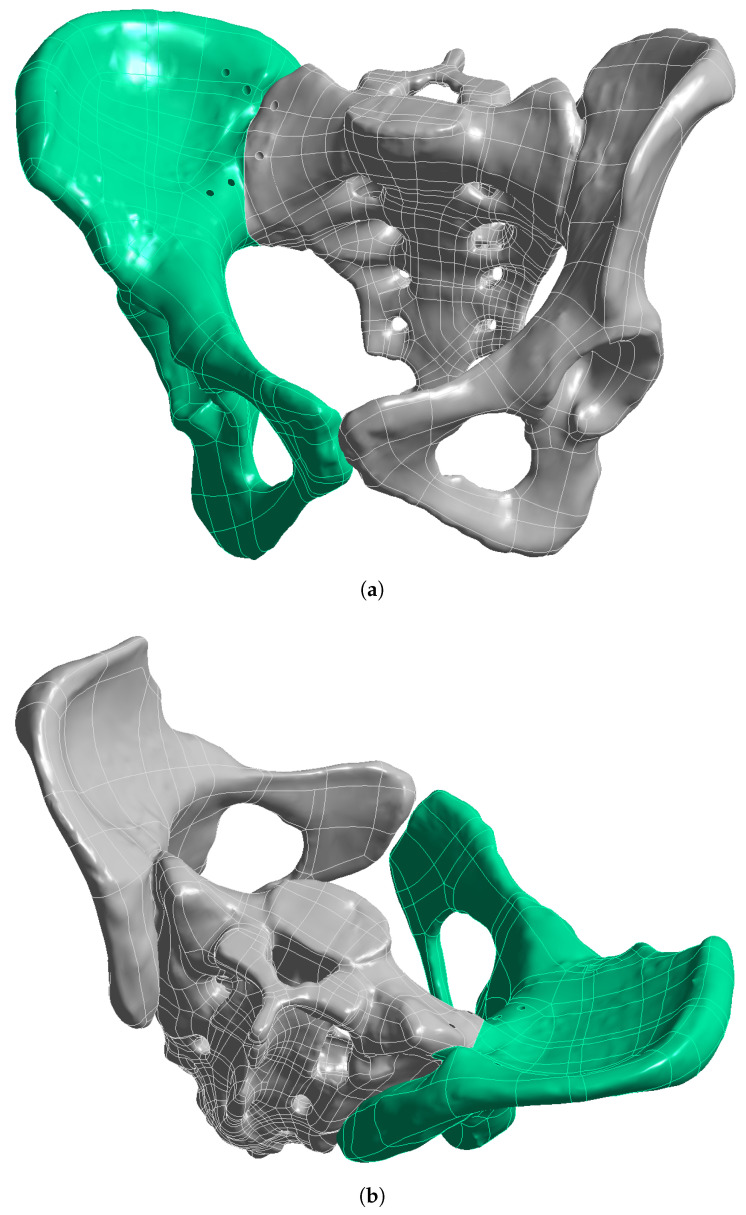
Tile type C1.2 pelvic ring fracture pattern showing the sacroiliac disruption and anterior ring fracture: (**a**) Anterior view, (**b**) Posterior view.

After reconstruction of the intact pelvis, a Tile C1.2 injury pattern was simulated by introducing a complete unilateral posterior separation of the sacrum from the right ilium through the right sacroiliac joint, while the contralateral hemipelvis remained intact. The corresponding anterior ring disruption was modeled at the level of the pubic symphysis, producing a combined rotational and vertical instability corresponding to a Tile type C1.2 lesion. A 1.0 mm fracture gap was introduced at both the posterior sacroiliac and anterior symphyseal disruption sites to simulate clinically relevant separation and to enable micromotion analysis, consistent with prior FEA studies on pelvic ring injuries [14,15] (see Figure 1).

**Figure 2:**
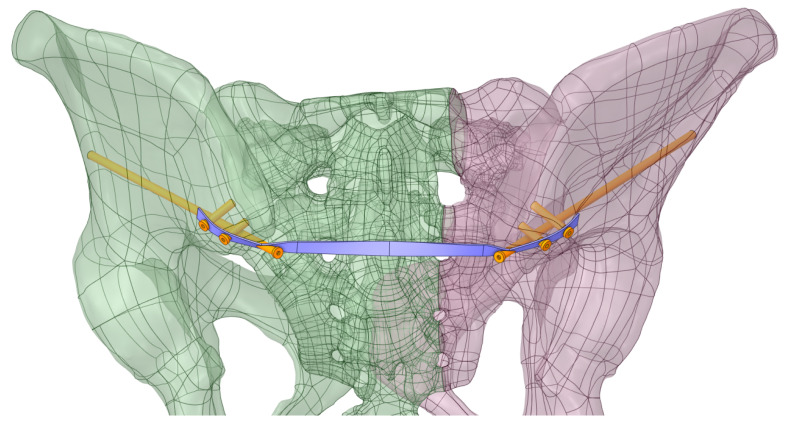
Transiliac plate construct spanning from one posterior iliac wing to the contralateral side across the sacrum.

**Figure 3:**
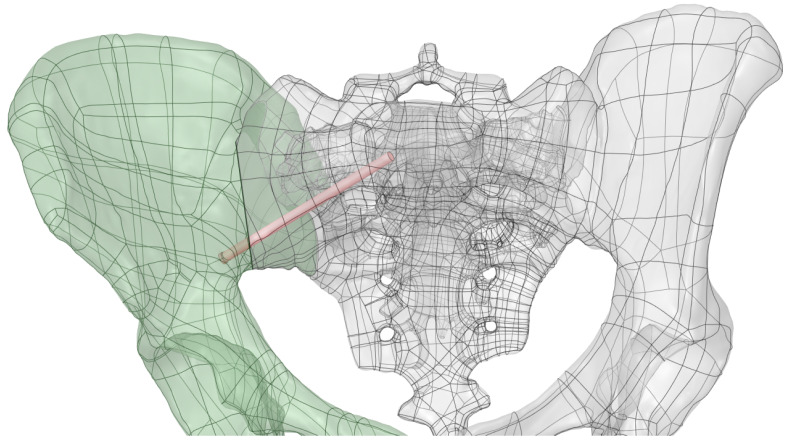
Iliosacral screw construct with one 6.5 mm cannulated screw positioned into the first sacral segment (S1).

**Figure 4:**
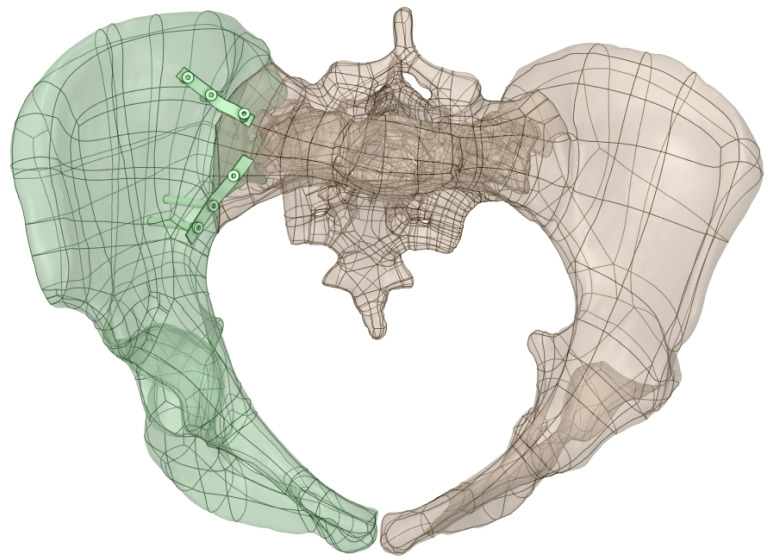
Anterior reconstruction plate construct with 3.5 mm plates along the sacroiliac joint and pubic region.

On this fractured model, three different fixation constructs were designed in SpaceClaim in accordance with manufacturer specifications and standard surgical techniques. The transiliac plate construct consisted of a 3.5 mm reconstruction plate (Auxein Inc., Doral, FL, USA) spanning from one posterior iliac wing to the contralateral side across the sacrum, with one screw inserted between the two plates of the iliac bone along the supra-acetabular canal, and two bicortical screws on each side, inserted perpendicular to it (Figure 2). The iliosacral screw construct comprised one partially threaded cannulated screw with a diameter of 6.5 mm and a length of 90 mm, with the threaded portion located within the sacral body, positioned from the iliac wing into the first sacral segment (S1) following the safe corridors described in the literature for percutaneous fixation (Figure 3). The anterior reconstruction plate construct included two 3.5 mm reconstruction plates molded along the anterior aspect of the sacroiliac joint arranged in a “V” shape, with one screw inserted into the sacral wing and two bicortical screws inserted into the iliac wing (Figure 4). For all three configurations, the positions of plates and screws were adjusted iteratively to avoid cortical perforation and to reproduce clinically used implant locations.

### 2.2. Material Properties and Mesh Creation

The material properties of the finite element models (pelvic bone and implants) were assigned according to values commonly reported in the literature [16,17] and are listed in Table 1. All materials were assumed to be homogeneous, isotropic and linearly elastic.

Cancellous bone was not modeled as a separate material; instead, a single equivalent material property was assigned to the pelvic bone to allow consistent comparative analysis between fixation constructs.

**Figure 5:**
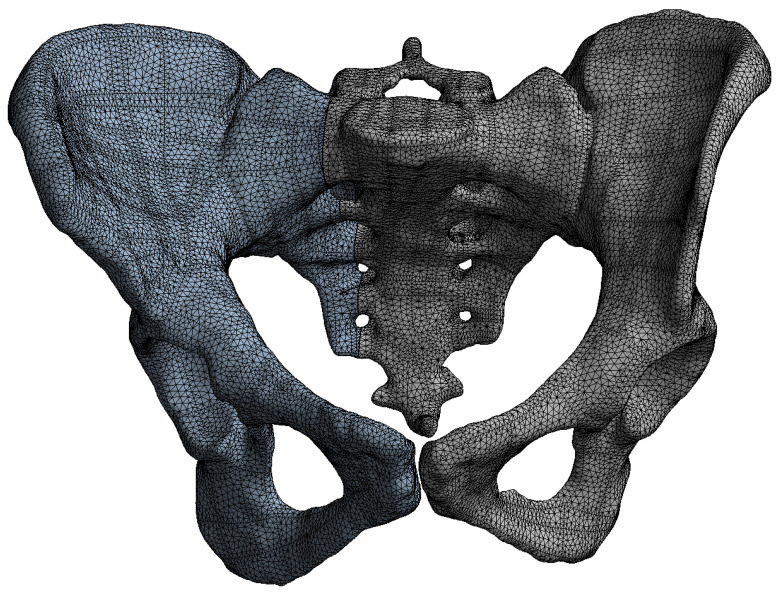
Tetrahedral mesh of the pelvic bone and implant models.

Tetrahedral finite elements were created by Ansys Mechanical on the pelvic bone structures and implants (Figure 5). The transiliac plate construct model was meshed with 332,856 elements and 598,432 nodes. The iliosacral screw construct model contained 310,214 elements and 552,108 nodes. The anterior reconstruction plate construct model was meshed with 318,672 elements and 571,285 nodes. A mesh convergence study was performed to ensure that further refinement did not significantly alter the results. The convergence criterion was defined as less than 5% change in peak von Mises stress and maximum displacement between successive mesh refinements. All three models satisfied this criterion at the reported mesh densities.

### 2.3. Boundary and Loading Conditions

The friction coefficient between the bone and implant surfaces was assigned a value of 0.3, consistent with values reported in prior pelvic finite element studies [16,18], except for the bone–screw interfaces which were modeled as fully bonded to simulate stable initial fixation. This fully bonded assumption represents ideal screw purchase and may overestimate interface strength; however, it was applied uniformly across all constructs to preserve comparative validity. Contact between fracture fragments was defined as frictionless to allow physiological micromotion.

**Figure 6:**
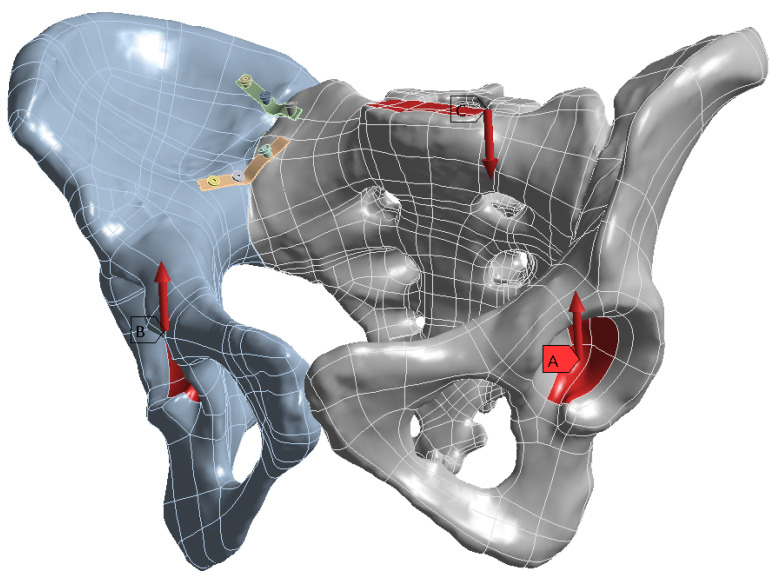
Force distribution and orientation applied to the sacral endplate (highlighted in red).

We performed two different tests with vertical loads of 400 N and respectively 800 N applied to the superior surface of the sacral endplate (Figure 6) to simulate partial weight bearing and full standing stance, respectively. These loads correspond to approximately 48% and 96% of body weight for the modeled 85 kg subject (833 N) and were selected to represent conservative partial and near-full weight-bearing conditions.

**Figure 7:**
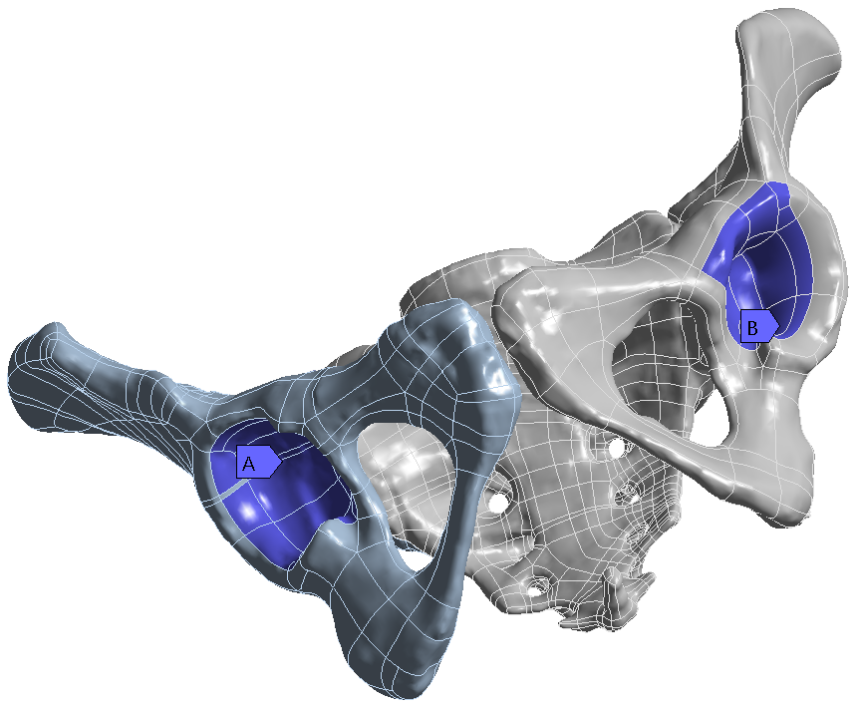
Fixed boundary conditions at the acetabular regions (highlighted in dark blue).

The acetabular regions of both hemipelves were fully constrained in all degrees of freedom (Figure 7). In finite element analysis, a fully constrained surface is one on which all degrees of freedom are restricted, meaning that the nodes on the surface cannot translate or rotate in any direction in response to the applied loads. This boundary condition simulates the support provided by the femoral heads during standing stance. This boundary condition was chosen to simulate double-limb stance and to ensure consistent comparison across constructs. While full constraint may overestimate global stiffness, it represents a commonly used approach in finite element pelvic studies, and its bias was applied uniformly across all three configurations. Similar acetabular constraints have been employed in previous pelvic finite element analyses [16,18,19].

## 3. Results

In this section, we present the outcomes of the finite element analyses for the three fixation constructs under partial (400 N) and full (800 N) vertical loading. The results are expressed as von Mises stresses in the implants, von Mises stresses in the pelvic bone, and overall pelvic displacement as a measure of construct stiffness. Von Mises stress is a scalar value representing the equivalent stress in a material, taking into account the combined effects of normal and shear stresses; it is commonly used to predict yielding and is also known as the equivalent or effective stress. In all contour plots presented in this section, the color scale ranges from blue to red, where blue represents the lowest values and red represents the highest values of the displayed quantity. This convention applies to both von Mises stress and total deformation maps.

### 3.1. Stresses and Deformation on the Transiliac Plate Construct

**Figure 8:**
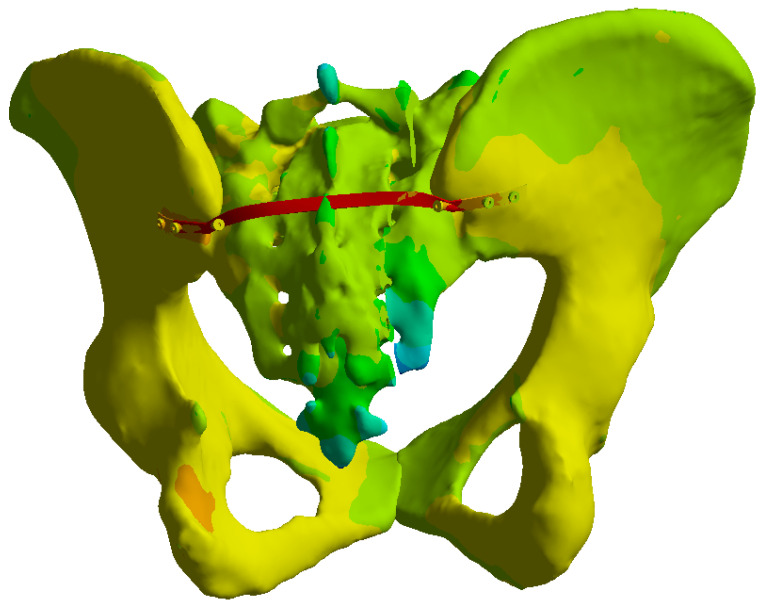
Von Mises stress distribution on the bone–implant system for the transiliac plate construct under 800 N loading.

**Figure 9:**
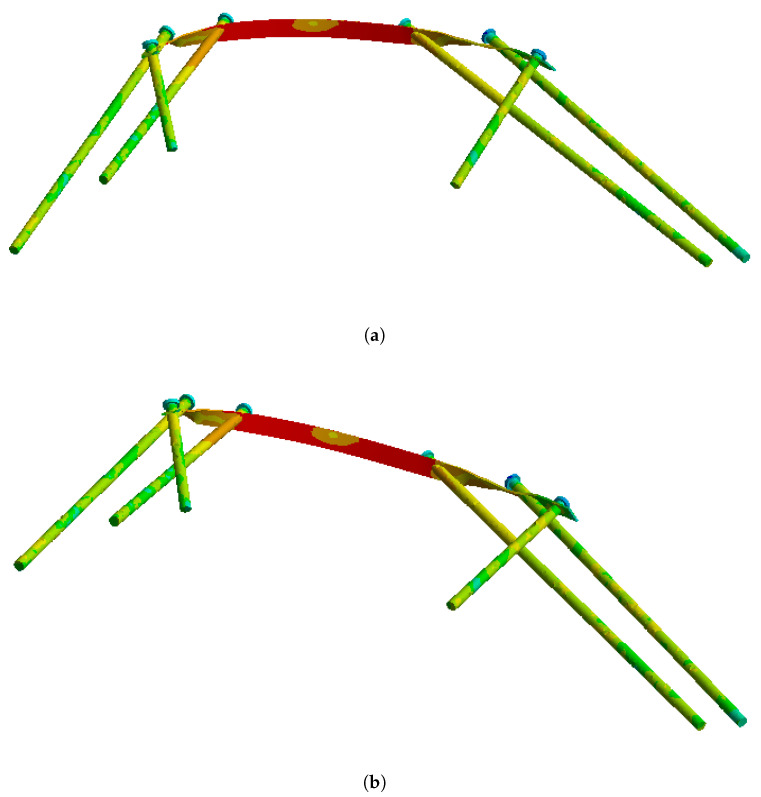
Von Mises stress distribution on the transiliac plate and screws under (**a**) partial (400 N) and (**b**) full (800 N) weight-bearing conditions.

The finite element simulations showed that peak von Mises stresses in the transiliac plate were located in the central portion bridging the sacrum as well as around the screw holes adjacent to the posterior fracture line (Figure 8). Under the 800 N loading condition, the maximum stress in the plate reached approximately 207 MPa, which remains below the yield strength of structural steel (250 MPa). The stress distribution along the plate was relatively homogeneous, with gradual transitions from highly loaded to moderately loaded regions. At the plate–screw junctions nearest the sacroiliac disruption, stress concentrations of 100–200 MPa were observed, while the peripheral portions of the plate experienced stresses below 100 MPa. The stress distribution on the implant elements alone is shown in Figure 9.

**Figure 10:**
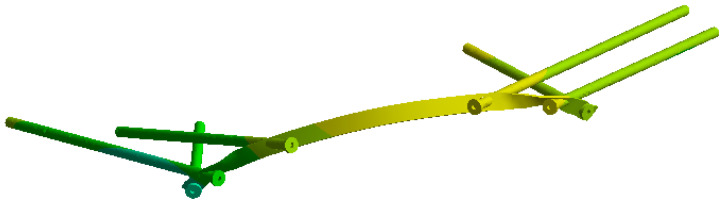
Total deformation of the transiliac plate construct under 800 N loading.

The total deformation analysis revealed that the transiliac plate construct exhibited the largest maximum displacement among the three constructs, with values of approximately 1.17 mm under 400 N and 1.38 mm under 800 N loading (Figure 10).

### 3.2. Stresses and Deformation on the Iliosacral Screw Construct

**Figure 11:**
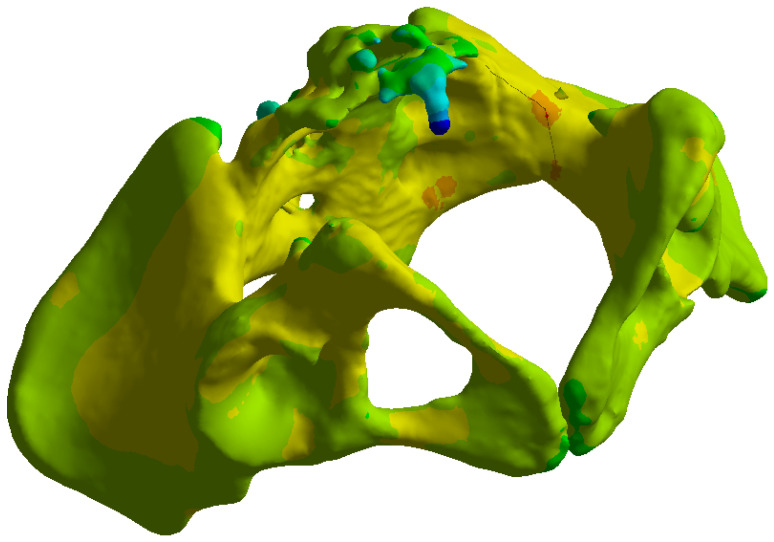
Von Mises stress distribution on the bone–implant system for the iliosacral screw construct under 800 N loading.

**Figure 12:**
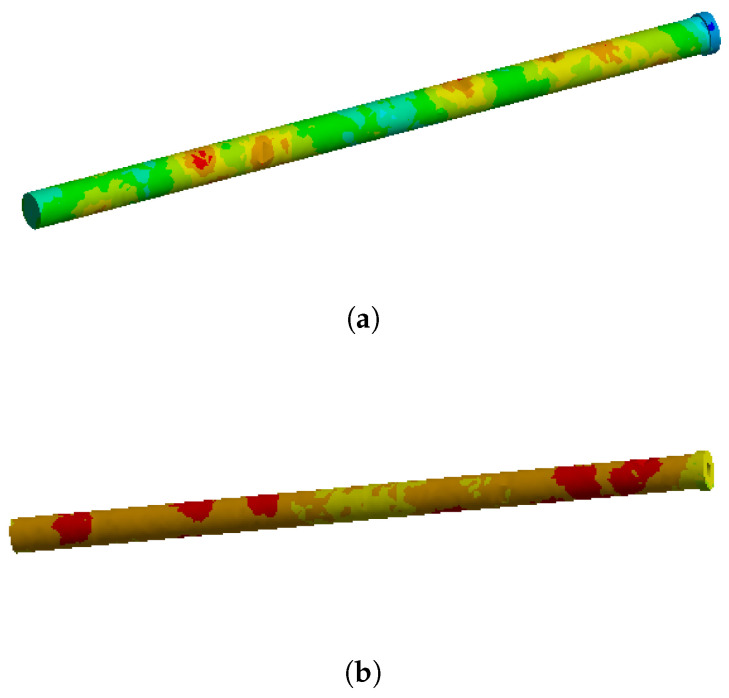
Von Mises stress distribution on the iliosacral screw under (**a**) partial (400 N) and (**b**) full (800 N) weight-bearing conditions.

In the iliosacral screw construct, stress concentrations were lower and more localized compared to the transiliac plate. The greatest von Mises stresses occurred around the screw threads within the sacral body and at the iliac entry points (Figure 11). At 800 N loading, the maximum stress in the screw reached 212 MPa, while the central shaft of the screw remained less loaded, indicating that the critical regions are the bone–implant interfaces rather than the screw body itself. The stress distribution showed marked heterogeneity, with sharp transitions between high-stress zones around the thread tips and low-stress zones along the smooth screw shafts. The stress distribution on the screw elements alone is shown in Figure 12.

**Figure 13:**
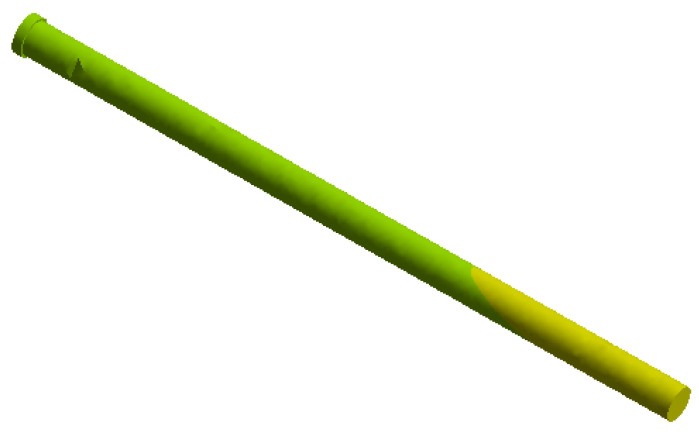
Total deformation of the iliosacral screw construct under 800 N loading.

The total deformation analysis showed that the iliosacral screw construct exhibited the smallest displacement among the three constructs, with maximum values of approximately 0.7 mm under 400 N and 1.15 mm under 800 N loading (Figure 13).

### 3.3. Stresses and Deformation on the Anterior Reconstruction Plates Construct

**Figure 14:**
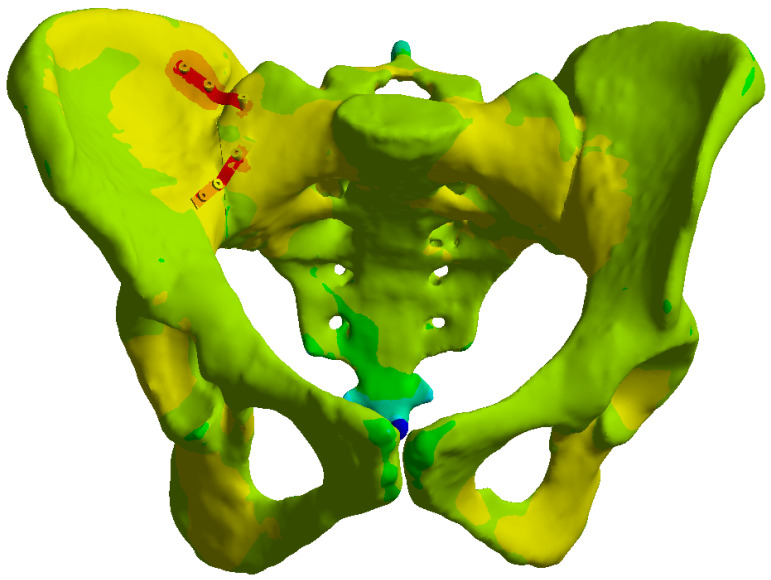
Von Mises stress distribution on the bone–implant system for the anterior reconstruction plate construct under 800 N loading.

**Figure 15:**
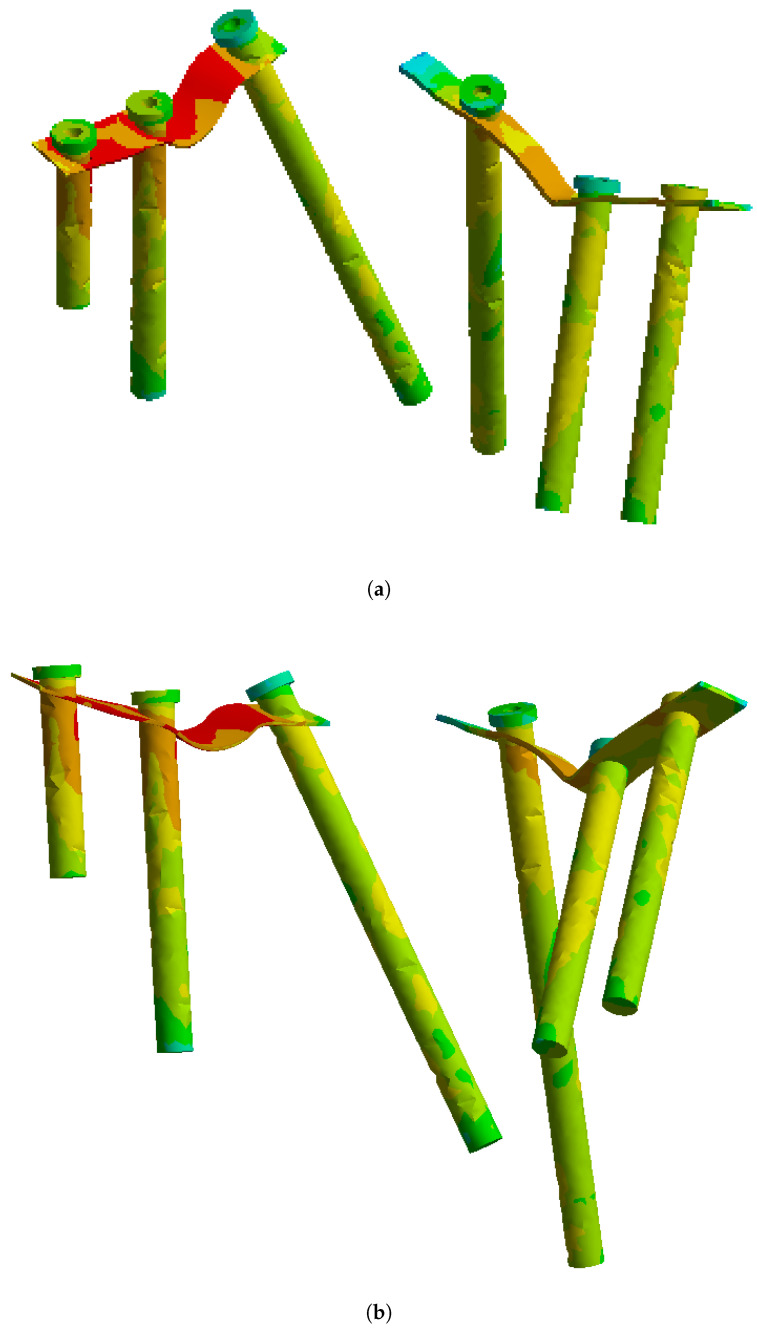
Von Mises stress distribution on the anterior reconstruction plates and screws under (**a**) partial (400 N) and (**b**) full (800 N) weight-bearing conditions.

For the anterior reconstruction plate construct, the greatest stresses were observed in the plates spanning the sacroiliac joint and at the screw holes adjacent to the joint line (Figure 14). Under full loading, the maximum von Mises stress was approximately 200 MPa, intermediate between the other two constructs. The distribution was less uniform than in the transiliac plate construct, with pronounced peaks at the sacroiliac level where the plates bridge the disruption. The stress distribution on the plate and screw elements alone is shown in Figure 15.

**Figure 16:**
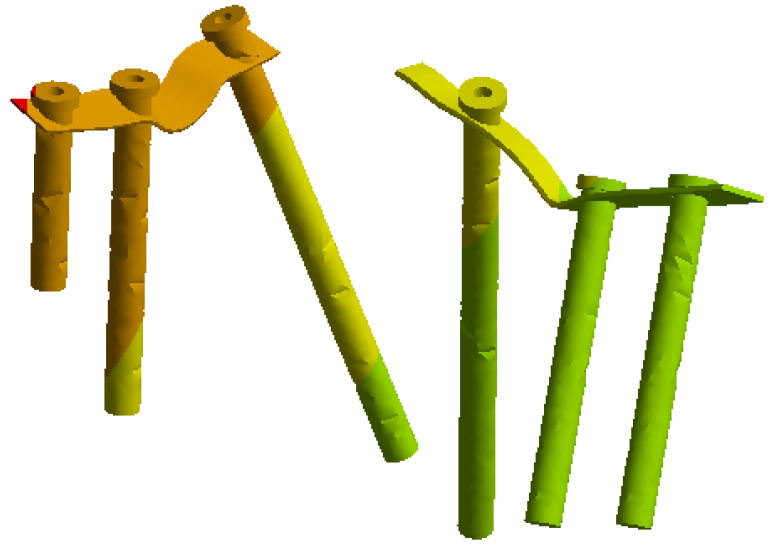
Total deformation of the anterior reconstruction plate construct under 800 N loading.

The total deformation analysis revealed that the anterior reconstruction plate construct showed intermediate displacement values, with maximum deformation of approximately 1.04 mm under 400 N and 1.76 mm under 800 N loading (Figure 16).

### 3.4. Stresses on Pelvic Bone

In all three constructs, bone stresses were mainly concentrated around the sacroiliac joint and the posterior sacral body. The transiliac plate construct showed the lowest peak stresses in the pelvic bone, with a maximum of approximately 70 MPa under 800 N loading (Figure 8). This stress was distributed over a relatively broad area of the dorsal sacrum and ipsilateral ilium, suggesting efficient load sharing between the implant and the surrounding bone.

The iliosacral screw construct generated higher and more focal bone stresses (Figure 11). Peak values near the screw threads in S1 and at the iliac entry points approached 95 MPa. These localized stress concentrations represent relative differences within the model and should not be interpreted as direct predictions of clinical failure, as bone quality was not explicitly modeled. The potential clinical implications of these stress patterns are addressed in Section 4.

The anterior reconstruction plate construct produced intermediate bone stress levels, with a maximum of about 85 MPa on the anterior surface of the sacroiliac joint (Figure 14). Stresses around the pubic ramus fracture were lower and spread over a wider area, consistent with the supportive role of the anterior ring.

### 3.5. Displacement of Pelvic Bone

Global displacement of the pelvic ring was used as the primary indicator of construct stiffness, defined as the maximum nodal displacement observed anywhere in the finite element model under applied load. This metric reflects the overall resistance of each fixation construct to vertical deformation and allows direct comparison between configurations under identical boundary and loading conditions. Construct stiffness was assessed indirectly through displacement under identical loading conditions rather than computed as a discrete load-to-displacement ratio, as the apparent stiffness varied with load magnitude due to contact nonlinearities in the model.

Under the 400 N load, the iliosacral screw construct exhibited the lowest global displacement, with a maximum value of 0.70 mm. The anterior reconstruction plate construct demonstrated intermediate displacement of 1.04 mm, while the transiliac plate construct showed the largest displacement at 1.17 mm.

**Figure 17:**
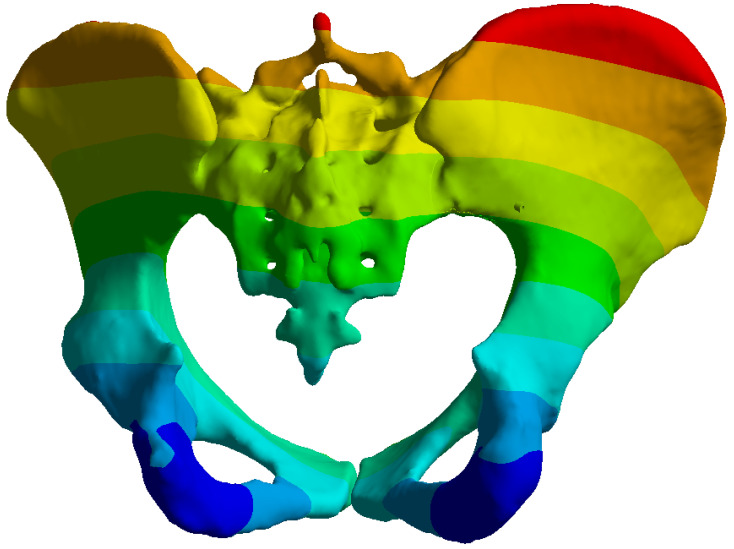
Total deformation of the bone and iliosacral screw construct under 800 N loading.

**Figure 18:**
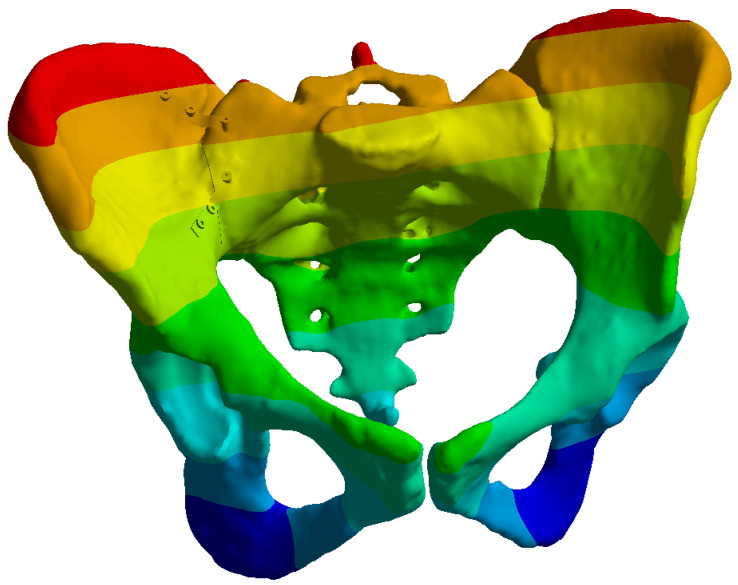
Total deformation of the bone and anterior reconstruction plate construct under 800 N loading.

**Figure 19:**
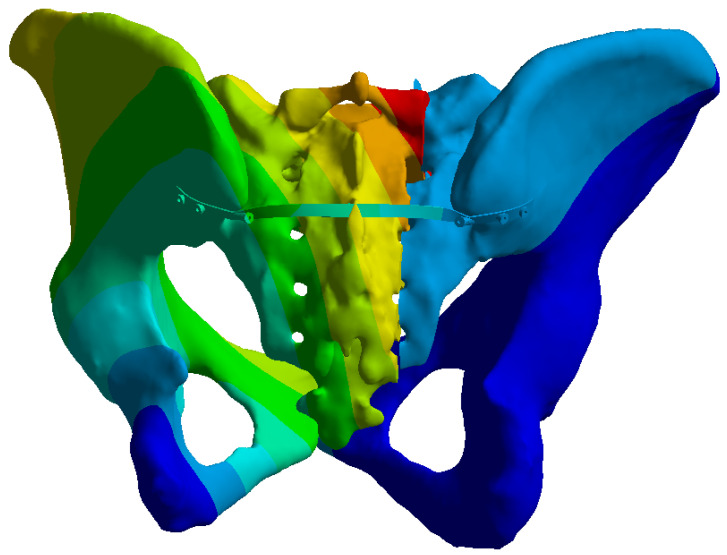
Total deformation of the bone and transiliac plate construct under 800 N loading.

When the load was increased to 800 N, the same ranking was preserved. Maximum global displacement reached 1.15 mm for the iliosacral screw construct, 1.76 mm for the anterior reconstruction plates, and 1.38 mm for the transiliac plate construct (see Figure 17, Figure 18 and Figure 19, respectively). These findings indicate that the iliosacral screw configuration provided the greatest resistance to vertical deformation, followed by the transiliac plate, while the anterior reconstruction plate construct allowed the largest global displacement under full loading.

Table 2 summarizes the maximum von Mises stress values in the implants, the maximum bone stress and the maximum displacement observed in each construct under both loading conditions.

## 4. Discussion

The aim of this finite element study was to compare the biomechanical behavior of three commonly used fixation constructs for Tile type C1.2 pelvic ring fractures: a transiliac plate, one iliosacral screw and two anterior reconstruction plates. The principal findings were that the iliosacral screw construct provided the greatest overall stiffness and the lowest pelvic displacement under vertical loading. The transiliac plate construct showed larger displacements, while the anterior reconstruction plate construct offered an intermediate level of stability.

These results are consistent with previous clinical and biomechanical reports highlighting the importance of robust posterior fixation for unstable pelvic ring injuries [4,6,11]. Iliosacral screws have gained popularity because they can be inserted percutaneously with minimal soft tissue disruption and reduced infection rates [7,8,9,10]. The present simulations demonstrate that the iliosacral screw construct provides excellent stiffness with the lowest displacement among the three constructs. However, the higher local stresses observed at the bone–implant interface could be problematic in osteoporotic patients or under repeated cyclic loading.

The transiliac plate construct effectively bridges both iliac wings and the sacrum, transforming the posterior pelvic ring into a continuous arch and thereby distributing loads more evenly. However, this configuration resulted in larger overall displacements compared to the other constructs. Clinically, transiliac plating has been associated with low rates of neurovascular complications when meticulous surgical technique is employed [11].

Stress distribution is an important indicator of potential hardware failure, and the stress magnitude is relevant for implant safety under the given loading conditions. In this study, the maximal vertical load applied to the sacral endplate was 800 N, representing approximately full body weight on both limbs during standing stance phase. None of the peak implant stresses exceeded the yield strength of the modeled structural steel (250 MPa), used in this study to approximate the properties of surgical-grade stainless steel (316L) implants, indicating that all three constructs would likely be safe under physiological loading in the early postoperative period. It should be emphasized that fatigue properties of the implant material were not modeled in this study, and the reported von Mises stress values should not be interpreted as clinical failure thresholds, but rather as comparative indicators of stress distribution under the modeled loading conditions. Nevertheless, the higher and more focal stresses observed in the iliosacral screw construct suggest a narrower margin of safety and a potentially greater susceptibility to fatigue failure or screw loosening with repeated loading.

Displacement at the fracture site is clinically relevant because excessive motion can impair bone healing and cause pain. The iliosacral screw construct exhibited displacements that were significantly lower than the other two constructs under full loading. The larger displacements observed in the transiliac plate and anterior reconstruction plate constructs may become clinically significant in patients who do not adhere to weight-bearing restrictions or who have compromised bone quality. Although no universally accepted threshold for allowable fracture-site micromotion exists in the literature for pelvic ring fractures, the displacements reported here are used as a comparative surrogate to rank construct performance under identical conditions.

Finite element analysis has been widely used to evaluate orthopaedic implants because it allows a controlled comparison of multiple configurations under standardized conditions. While our methodology follows established practices, several limitations should be acknowledged. First, pelvic bone was modeled as a homogeneous and isotropic material, without separate representation of cancellous bone, which may influence absolute stress magnitudes at the bone–implant interface. Second, the finite element model did not distinguish between cortical and cancellous bone; instead, the entire pelvic bone was assigned a single equivalent material property to simplify the analysis. While this approach is commonly used, it may overestimate stress concentrations, particularly around screw threads where cancellous bone typically offers a more compliant mechanical environment. However, this simplification was necessary to allow consistent comparative evaluation across all fixation constructs. Third, the analyses were limited to static vertical loading conditions of 400 N and 800 N. In vivo, the pelvic ring is subjected to combined axial, shear, and torsional loads, particularly during gait and transitional movements. Fourth, the acetabular regions were fully constrained to simulate standing support, a boundary condition that likely overestimates absolute construct stiffness but preserves the relative comparison between fixation methods. Fifth, ligamentous and muscular structures of the pelvic ring were not included, although they play a significant role in physiological load transfer and stability. In particular, the absence of cyclic loading and ligamentous structures should be considered when extrapolating these findings to early weight-bearing or long-term fixation durability. Finally, the simulations were based on a single anatomical model and therefore do not capture interindividual variability in pelvic morphology or bone quality. While the selected anatomy is representative of a typical adult male pelvis, it does not capture sex-based or individual variability in pelvic geometry.

Among these limitations, the omission of ligamentous and muscular structures is most likely to affect the absolute displacement values and could differentially influence constructs that rely on posterior tension band mechanics, such as the transiliac plate. The use of homogeneous bone properties is unlikely to alter the relative ranking of constructs but may affect the absolute magnitude of bone–implant interface stresses, particularly for the iliosacral screw. The fully bonded screw–bone assumption represents ideal fixation and may underestimate loosening risk in clinical practice; a sensitivity analysis comparing bonded and frictional screw interfaces would be informative in future studies. Conversely, the boundary conditions and static loading simplifications are expected to bias all constructs equally and are therefore unlikely to change the comparative ranking.

Despite these limitations, the comparative nature of the study remains valid, as all three constructs were assessed under identical assumptions and loading conditions. Future work should include patient-specific models with heterogeneous material properties, as well as experimental validation using cadaveric specimens or physical models. Cyclic loading protocols would also be valuable to assess fatigue behavior and long-term stability of the constructs.

Although direct experimental validation was not performed in this study, the material properties, loading conditions and boundary conditions employed are consistent with those used in previously validated pelvic finite element models [16,18]. The displacement patterns and stress magnitudes observed in the present study are broadly comparable to those reported by Hu et al. [12], who analyzed similar posterior fixation strategies and found comparable stress ranges in pelvic bone and implants. Nonetheless, future work should seek to validate the present models against cadaveric testing or clinical imaging data.

One of the strengths of our study is the high mesh density used for all three models, which enhances the accuracy of stress and displacement predictions. The mesh convergence analysis confirmed that further refinement did not significantly alter the results, lending confidence to the numerical outcomes.

Within the constraints of our models, the present study suggests that iliosacral screw fixation provides the greatest resistance to displacement for Tile type C1.2 pelvic ring fractures under the tested conditions, while the transiliac plate showed larger displacements.

Combined fixation strategies that leverage the advantages of multiple constructs may be considered in selected cases to balance mechanical stability and surgical invasiveness.

## 5. Conclusions

This finite element study compared the biomechanical behavior of three posterior fixation constructs for Tile type C1.2 pelvic ring fractures under simulated partial and full weight-bearing conditions. Among the tested constructs, the iliosacral screw configuration demonstrated the greatest resistance to displacement and therefore the highest construct stiffness, while the transiliac plate allowed the largest global displacement. The anterior reconstruction plates exhibited intermediate mechanical performance.

The observed stress distributions indicate that the iliosacral screw concentrates load at the bone–implant interface, whereas transiliac plating promotes more distributed load transfer across the posterior pelvic ring. These differences reflect distinct mechanical strategies rather than predictors of clinical failure. Because the model incorporated simplified material properties, static loading, and a single anatomical geometry, the results should be interpreted as a comparative assessment of construct behavior under controlled conditions rather than as definitive clinical guidance.

Despite these limitations, the findings provide useful biomechanical insight into the relative performance of commonly used fixation strategies for unstable pelvic ring injuries and may support surgical decision-making when combined with patient-specific factors such as bone quality, soft tissue condition, and associated injuries. Further experimental and clinical studies are required to validate these observations and to evaluate construct performance under cyclic and multiaxial loading.

## Figures and Tables

**Table 1:** Physical characteristics for finite element models.

Material	Young’s Modulus (MPa) ^1^	Poisson’s Ratio ^2^
Cortical bone	$1.89\times 10^{4}$	0.3
Structural steel ^3^ (implants)	$2.0\times 10^{5}$	0.3

^1^ Young’s modulus is a measure of a material’s stiffness and is used to model the elastic behavior of materials such as metals and bone. ^2^ Poisson’s ratio is a measure of a material’s lateral contraction and is used to model the deformation of materials in response to applied loads. ^3^ Structural steel material properties (E = 200 GPa, ν = 0.3) were used to approximate surgical-grade stainless steel (316L) implants, as the elastic modulus and Poisson’s ratio are closely comparable.

**Table 2:** Maximum von Mises stress in implants and bone, and maximum pelvic displacement for the three constructs under partial (400 N) and full (800 N) weight-bearing conditions.

	Max Implant Stress (MPa)	Max Bone Stress (MPa)	Max Global Displacement (mm)
**Transiliac plate**			
Partial weight bearing (400 N)	194	65	1.17
Full weight bearing (800 N)	207	70	1.38
**Iliosacral screw**			
Partial weight bearing (400 N)	157	71	0.70
Full weight bearing (800 N)	212	95	1.15
**Anterior reconstruction plates**			
Partial weight bearing (400 N)	195	55	1.04
Full weight bearing (800 N)	200	85	1.76

## Data Availability

The data presented in this study are available on request from the corresponding author due to privacy or ethical restrictions.
